# A Comprehensive Analysis of the Effect of SIRT1 Variation on the Risk of Schizophrenia and Depressive Symptoms

**DOI:** 10.3389/fgene.2020.00832

**Published:** 2020-07-31

**Authors:** Dandan Wang, Wei Tang, Junxiong Zhao, Weixing Fan, Yi Zhang, Chen Zhang

**Affiliations:** ^1^Shanghai Mental Health Center, Shanghai Jiao Tong University School of Medicine, Shanghai, China; ^2^Department of Psychiatry, The Affiliated Kangning Hospital of Wenzhou Medical University, Wenzhou, China; ^3^Department of Psychiatry, Jinhua Second Hospital, Jinhua, China

**Keywords:** schizophrenia, depression, SIRT1, CDSS, association, polymorphism

## Abstract

Depressive symptoms could be considered a mutual manifestation of major depressive disorder and schizophrenia. Rs3758391 is a functional locus of Sirtuin (SIRT1) involving depression etiology. In this study, we hypothesized that the SIRT1 SNP rs3758391 might be a hazard for schizophrenia pathogenesis, especially related to the appearance of depressive symptoms. We recruited 723 healthy controls and 715 schizophrenia patients, the occurrence of psychotic and depressive symptoms was evaluated by Calgary Depression Scale (CDSS) and PANSS. Meanwhile, qt-PCR was used to detect the mRNA levels of SIRT1 in peripheral blood of 197 olanzapine monotherapy schizophrenia patients. 45.6% of schizophrenia patients had depressive symptoms. In the patient group, mRNA levels of patients with depressive symptoms were significantly lower than those without depressive symptoms (*P* < 0.01). CDSS scores of schizophrenia patients with different rs3758391 genotypes were significantly different (*P* < 0.01). *Post hoc* comparisons indicated that the CDSS scores of rs3758391 C/C and C/T carriers were higher than those of T/T carriers (Ps < 0.01). In the occipital cortex, our eQTL analysis showed that there was a clear correlation between rs3758391 and the SIRT1 mRNA levels. Our preliminary findings provide suggestive evidence that SIRT1 makes schizophrenia patients more prone to depressive symptoms. This SNP might be a biomarker of depression in schizophrenia.

## Introduction

Schizophrenia is a heterogeneous group of syndromes whose main symptoms mainly involve multiple dimensions, including cognitive dysfunction, negative symptoms, and positive symptoms. And major depressive disorder (MDD) is another common serious mental illness, mainly manifested by symptoms such as inability to concentrate, loss of interest, and low mood. In clinical practice, depressive symptoms commonly accompany schizophrenia (Green et al., 2003). Depressive symptoms could be considered to be mutual manifestations of both schizophrenia and MDD (Chen et al., 2017). Studies have shown that about 50% of patients with schizophrenia have comorbid depression (Buckley et al., 2009). Although the pathophysiology of both of these mental disorders remains unknown, numerous family studies have highlighted the crucial role of genetic factors in the pathogenesis in MDD and schizophrenia (Zhang et al., 2016a). Notably, a good deal of genome-wide association research has uncovered overlapping genetic risk factors shared by patients with either disorder (Lee et al., 2013; Xiao et al., 2018).

Sirtuin (SIRT1) is an important nicotinamide-dependent protein deacetylase in the sirtuin protein family. The function of SIRT1 involves many aspects, and the functions that have been discovered so far mainly include regulating cell survival and apoptosis and inhibiting the stress-induced inflammatory response (Rahman and Islam, 2011). Additionally, SIRT1 also plays an important role in regulating biological rhythm and transducing dopaminergic signals (Kim et al., 2016). Subsequently, genetic studies in Eastern Asian populations (mainly Japanese and Chinese Han populations) indicated that the SIRT1 gene is associated with schizophrenia (Wang et al., 2015). Therefore, at least in Asian populations, SIRT1 variation is likely to contribute to the risk of schizophrenia.

Lately, a whole-genome sequencing experiment containing thousands of homogeneous Chinese samples identified one locus near SIRT1 that is significantly associated with MDD (CONVERGE consortium, 2015). Then our further study found that the expression of SIRT1 in the peripheral blood of MDD patients was significantly downregulated at the mRNA levels compared with that in the blood of healthy subjects (decreased by 37%; Luo and Zhang, 2016). Large-scale MDD expression data have further confirmed this finding (Jansen et al., 2016). Recently, in the Han Chinese population, we discovered a functional locus, rs3758391, in SIRT1 related to the etiology of MDD (Tang et al., 2018). The rs3758391 (T/C) is a gene promoter of the SIRT1 gene. And the critical functions of this SNP in the pathophysiology of human diseases are mainly manifested in the C variation might destroy the p53-binding sequence and affect the expression of SIRT1 (Naqvi et al., 2010; Hu et al., 2015). And our previous work also indicated that low plasma SIRT1 levels in schizophrenia patients are associated with depressive symptoms (Fang et al., 2019). Based on the above discoveries, we hypothesized that the SIRT1 SNP rs3758391 variation might be one of the causes of schizophrenia, especially its depressive symptoms.

In this research, we used public databases to explore the differences in SIRT1 expression in the brains of schizophrenic patients and healthy controls. Later, we attempted to identify the association of SIRT1 mRNA and the rs3758391 polymorphism with susceptibility to schizophrenia and associated depressive symptoms. Finally, in order to detect the effect of rs3758391 polymorphism on brain SIRT1 mRNA expression, we used an available database for eQTL analysis.

## Materials and Methods

### Subjects

Seven hundred and fifteen schizophrenia patients were recruited from mental health institute in Eastern China (Shanghai Mental Health Center, Affiliated Kangning Hospital, Wenzhou Medical University, and Jinhua Second Hospital), the inclusion criteria are in accordance with our previous publications and are as follows (Zhang et al., 2011; Cai et al., 2015; Wang et al., 2016; Zhang et al., 2018): (1) illness course less than 5 years; (2) with a stable condition over 6 months; (3) junior high school education or above; (4) not treated with a mood stabilizer or antidepressant; and (5) total scores for PANSS under 60 Patients who with other psychiatric disorders, had a severe physical disease, with substance dependence or abuse, are pregnant or nursing women were all excluded. Moreover, mRNA samples were obtained from 198 patients who treated with olanzapine monotherapy. The 723 healthy subjects were included as controls, and the detailed recruitment requirements were based on our previous studies (Zhang et al., 2017).

The Institutional Review Boards of Jinhua Second Hospital and other related institutions reviewed and approved this research. We obtained the written informed consent of all participants and strictly followed the experimental guidelines in the Declaration of Helsinki.

### Evaluation

The psychiatric symptoms and the severity of depression were evaluated by the PANSS and the Calgary Depression Scale (CDSS). Patients were defined as having significant depression if they had a CDSS score of 7 or higher (Peitl et al., 2017).

### Analysis of Brain SIRT1 Expression

The genetics and expression data platform for schizophrenia research named SZDB database^1^ were used to compare the SIRT1 expression between case and controls (Wu et al., 2017).

### RNA Preparation and Quantitative Real-Time Polymerase Chain Reaction

The RNA preparation and quantitative real-time polymerase chain reaction (qRT-PCR) were performed as our previous work (Luo and Zhang, 2016).

### Genotyping

The SNP rs3758391 was genotyped as our previous work has described (Zhang et al., 2014).

### PGC Data Analysis

The Psychiatric Genomics Consortium (PGC^2^) database was used to find out the association of rs3758391 polymorphism in schizophrenia. The more detailed description of this database sees our previous article (Zhang et al., 2016a).

### Brain eQTL Analysis

The brain eQTL database^3^ (Ramasamy et al., 2014) were utilized for eQTL analysis of the rs3758391 polymorphism in the brain.

### Statistical Analysis

We used the SHEsis software to compare the genotype and allele distribution in the case-control study. The Hardy-Weinberg equilibrium (HWE) was calculated by Haploview 4.2.

The ANCOVA was used to compare the CDSS scores between groups with different genotypes. When comparing the difference in SIRT1 mRNA expression between groups, we used ANCOVA to further controlled the potential covariates including age, gender, duration of olanzapine treatment, and olanzapine daily dosage. In addition, Pearson’s correlation analysis was conducted to explore the correlation between the levels of SIRT1 mRNA expression and CDSS scores in the patients. SPSS 17.0 was used for statistical analyses. All tests were two-tailed, with significance set to *p* < 0.05.

## Results

**FIGURE 1:**
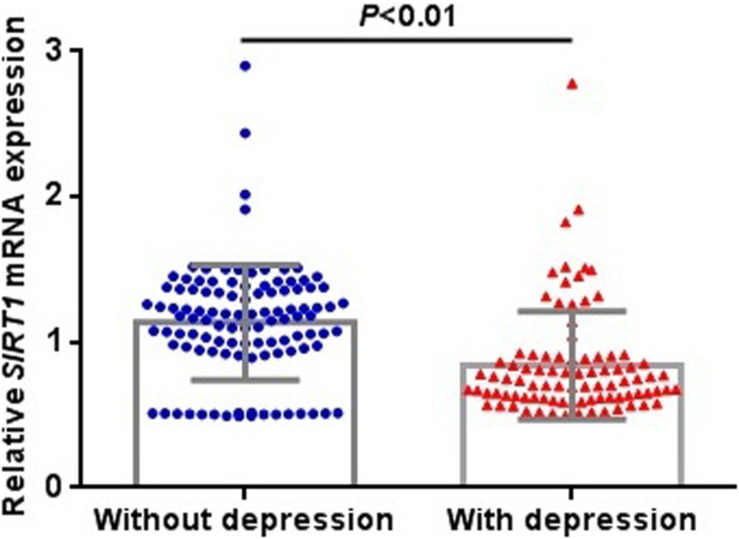
Differential expression of SIRT1 mRNA in brain between patients with schizophrenia and healthy controls. Each bar represents the average level of SIRT1 mRNA expression. Error bars represent the standard deviation of the mean value. Data was extracted from the SZDB database (http://www.szdb.org/).

We extracted the data for SIRT1 mRNA expression in the brain from the SZDB database. But we failed to find any significant differences in SIRT1 mRNA expression in the striatum, prefrontal cortex, or hippocampus of the case and control groups (Figure 1). At the molecular level, the distribution of rs3758391 genotypes in healthy controls and schizophrenia patients all established HWE. Table 1 presented comparisons of the allele frequency and genotype of the rs3758391 SNP in schizophrenia and healthy controls but found no difference. Next, we also found no significant association between the rs3758391 polymorphism and schizophrenia in the PGC database (Supplementary Figure S1).

**TABLE 1:** Distribution of rs3758391 genotype and allele in schizophrenia and controls, and schizophrenia with or without depression.

**SNP**		**Genotype, *N* (%)**				**Allele, *N* (%)**			
**rs3758391**	**N**	**C/C**	**C/T**	**T/T**	**P**	**C**	**T**	**P**	**OR (95%CI)**
Schizophrenia	715	25 (3.5)	207 (29.0)	483 (67.6)	0.18	257 (18.0)	1173 (82.0)	0.11	1.17 (0.96–1.42)
Controls	723	15 (2.1)	198 (27.4)	510 (70.5)		228 (15.8)	1218 (84.2)		
Schizophrenia									
With depression	326	13 (4.0)	110 (33.7)	203 (62.3)	0.022	136 (20.9)	516 (79.1)	0.009	1.43 (1.09–1.88)
Without depression	389	12 (3.1)	97 (24.9)	280 (72.0)		121 (15.6)	657 (84.4)		

**FIGURE 2:**
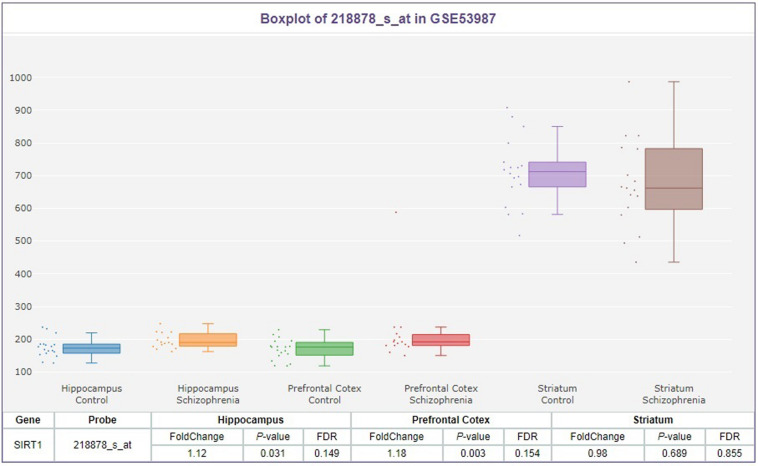
Expression levels of SIRT1 mRNA in peripheral blood in schizophrenia patients with or without depression. SIRT1 mRNA was normalized to that of GAPDH.

After evaluating 715 schizophrenia patients, according to the CDSS, 326 patients (45.6%) were determined to have depressive symptoms. In the allele frequency and genotype comparison of the rs3758391 polymorphism between schizophrenia with and without depression, we observed no significant correlation between this SNP and depression in schizophrenic patients (*P* = 0.022). But we found that the T allele frequency of rs3758391 in schizophrenia patients with depression is significantly lower than that of schizophrenia patients without depression (OR = 1.43, 95% CI: 1.09–1.88, and *P* = 0.009). In addition, we measured the peripheral mRNA expression of SIRT1 in 89 schizophrenia patients with depression and 108 without depression. The primary clinical information of these two cohorts was well matched (Supplementary Table S1). Figure 2 suggests that SIRT1 mRNA levels of schizophrenia patients with depression were significantly lower than those of schizophrenia without depression (*P* < 0.01). Consistent with our previously published results (Fang et al., 2019), the present study also suggested that the CDSS score was negatively correlated with SIRT1 mRNA levels (*r* = −0.328, *P* < 0.01).

**FIGURE 3:**
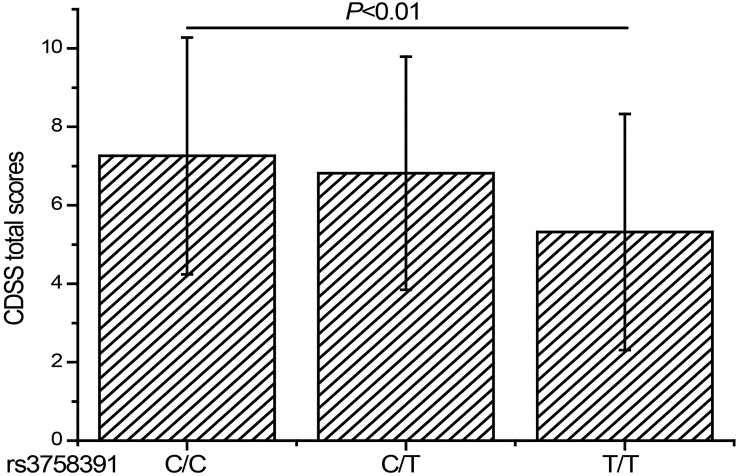
CDSS scores of schizophrenia patients classified according to the genotypes of rs3758391 polymorphism. Each column represents the mean ± SD. C/C (*n* = 25), C/T (*n* = 207), and T/T (*n* = 483).

**FIGURE 4:**
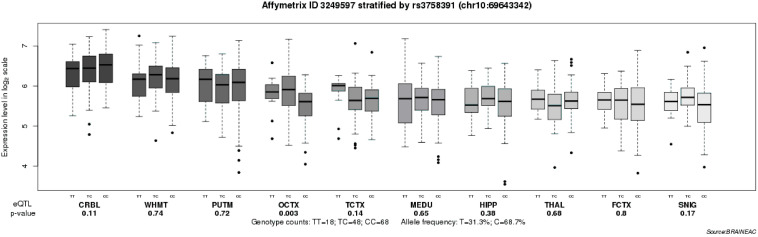
Association of rs3758391 with SIRT1 mRNA expression levels in ten brain regions (Affymetrix ID 3249597; http://peana-od.inf.um.es:8080/ UKBECv12/). SNIG, substantia nigra; PUTM, putamen (at the level of the anterior commissure); MEDU, the inferior olivary nucleus (sub-dissected from the medulla); THAL, thalamus (at the level of the lateral geniculate nucleus); OCTX, occipital cortex; HIPP, hippocampus; FCTX, frontal cortex; TCTX, temporal cortex; WHMT, intralobular white matter; and CRBL, cerebellar cortex.

To further explore the relationship between the rs3758391 polymorphism and depressive symptoms in patients with schizophrenia, we compared the CDSS scores of different genotypes of the rs3758391 polymorphism and found a significant difference (*P* < 0.01; Figure 3). *Post hoc* comparison showed that the CDSS score of rs3758391 C/C and C/T carriers were higher than that of T/T carriers (Ps < 0.01). Then, our eQTL analysis suggested a significant association between rs3758391 and SIRT1 mRNA expression in the occipital cortex (Figure 4), and SIRT1 mRNA expression levels in carriers with C/C genotypes were significantly lower than carriers with T/T genotype. However, we did not find this difference in the peripheral blood samples.

## Discussion

Sirtuin was identified as an MDD risk gene in a large-scale Chinese GWAS (CONVERGE consortium, 2015). Our recent meta-analysis indicated that among the Han Chinese population, the SIRT1 rs3758391 polymorphism confers susceptibility to MDD (Tang et al., 2018). Schizophrenia and MDD are distinct categorical diagnoses, but there is evidence of molecular genetic mechanisms overlap between the disorders (Schulze et al., 2014). Therefore, we aimed to identify the impact of SIRT1 rs3758391 polymorphism on the genetics and symptoms of schizophrenia in this research.

In this study, we found neither the association between SIRT1 mRNA expression in the brain and schizophrenia nor the significant effect of the rs3758391 polymorphism on susceptibility to schizophrenia in PGC GWAS. It suggests that the SIRT1 SNP rs3758391 probably does not confer an increased risk of schizophrenia. We further stratified the schizophrenia patients as either subject with or without depression based on the CDSS evaluation. Our study demonstrated that SIRT1 mRNA expression is significantly downregulated in schizophrenia with depression compared with that in those without depression, and patients with the C allele of rs3758391 have more severe symptoms of depression, which means the SNP rs3758391 might be the determinant of depressive symptoms in schizophrenia. To shed light on the impact of rs3758391 polymorphism on SIRT1 mRNA expression, an eQTL analysis was performed, which discovered that rs3758391 is significantly associated with SIRT1 mRNA expression in the occipital cortex. These consistent results indicated that genetic variation resulting from the rs3758391 polymorphism might lead to the dysregulation of SIRT1 mRNA expression and exert a significant influence on the occurrence and development of depressive symptoms in schizophrenia.

Sirtuin is responsible for oxidative respiration and cellular survival and therefore is related to inflammation, glucose stasis, apoptosis, and aging (Kauppinen et al., 2013). Researchers demonstrated that chronic stress could increase the risk of depression-like behavior by reducing SIRT1 activity by establishing a mouse model of depression (Abe-Higuchi et al., 2016). However, SIRT activators can improve such phenotypes (Hurley et al., 2014). For humans, convergent genetic evidence has verified the important role of SIRT1 in the etiology of MDD (CONVERGE consortium, 2015; Luo and Zhang, 2016). Our recent work provided evidence that suggests that the low plasma SIRT1 concentration might cause depression in patients with schizophrenia (Fang et al., 2019), which is also consistent with the results shown in the present study. There is evidence showing that SIRT1 can promote the secretion of IL-6, which exerts a significant influence on the regulation of the inflammatory response (Tang et al., 2017). Interestingly, we found a significant increase in IL-6 mRNA levels in patients with MDD (Zhang et al., 2016b). In summary, we speculated that the effect of SIRT1 on the development of depressive symptoms in schizophrenia might be achieved by regulating the inflammatory response. Our previous study indicated that the pathophysiology of MDD might involve immune dysfunction (Zhang et al., 2016c). Thus, a question has naturally arisen regarding whether such a mechanism underlies the development of depressive symptoms in schizophrenia. Further investigations are required for clarification.

The SNP rs3758391 is a gene promoter located at the p53-binding site of the SIRT1 gene (Naqvi et al., 2010). This study indicated that rs3758391 is likely to affect the severity of depressive symptoms in schizophrenia. Our eQTL results showed that rs3758391 is closely related to the mRNA expression of SIRT1 in the occipital cortex and that rs3758391 C/C carriers have significantly lower SIRT1mRNA expression in the occipital cortex than T/T carriers. Emerging evidence suggested that the pathogenesis of MDD is related to the dysfunction of the occipital cortex mediated by the complement factor H (CFH), an important inflammatory molecule (Maciag et al., 2010; Zhang et al., 2016c). Thus, we assumed that inflammation might be the basis of the mechanism of the occipital cortex involved in the depressive symptoms in schizophrenia; however, this conclusion requires further investigations. The HapMap project has documented that the overall average heterozygosity frequency of the rs3758391 polymorphism is 50%. In the NCBI database, the C allele of rs3758391 is found at a frequency of 15.1% in HCB and a frequency of 73.0% in CEU. This implies that rs3758391 might be an ethnically-specific polymorphism. Thus, our analysis should be replicated in Caucasian populations.

Despite the promising implications of our results, there are several major limitations should be noted. First and foremost, we analyzed the effect of only one SNP in SIRT1 on schizophrenia. This means that further analysis of more SIRT1 gene variants is needed to verify our results. Second, the patients included in the group were all treated with antipsychotics and were stable for more than 6 months. As is well-known, antipsychotic treatment is likely to bias the symptomatology (Zhang et al., 2017). Finally, due to the inherent characteristics of cross-sectional studies, we failed to determine whether SIRT1 levels have changed before the onset of depressive symptoms in schizophrenia. In summary, our research was only exploratory and preliminary.

To the best of our knowledge, this study explored the relationship between SIRT1 rs3758391 polymorphism and schizophrenia for the first time. In this study, we performed a comprehensive investigation to detect the potential link between rs3758391 and schizophrenia. Our preliminary findings provide suggestive evidence that SIRT1 confers susceptibility to depressive symptoms in schizophrenia. The SNP rs3758391 functionally affects the severity of depressive symptoms in schizophrenic patients. This SNP probably served as one of the biomarkers of schizophrenia depression. Our work is a commendable attempt to promote the diagnosis and treatment of different subtypes of schizophrenia, which provides useful information that improves the understanding of the genetic mechanism of depressive symptoms in schizophrenia. These findings should be further validated by more extensive sample studies in the broader population.

## Data Availability Statement

The raw data supporting the conclusions of this article will be made available by the authors, without undue reservation, to any qualified researcher.

## Ethics Statement

The studies involving human participants were reviewed and approved by Institutional Review Boards of Jinhua Second Hospital. The patients/participants provided their written informed consent to participate in this study.

## Author Contributions

CZ and DW contributed to the overall design of the study. JZ and WF wrote the protocol for the genotyping. JZ, DW, WF, WT, and YZ got involved sample collection. JZ and CZ undertook the statistical analysis and interpretation of data. CZ and DW wrote the manuscript. All authors contributed to the article and approved the submitted version.

## Conflict of Interest

The authors declare that the research was conducted in the absence of any commercial or financial relationships that could be construed as a potential conflict of interest.

## Footnotes

1 http://www.szdb.org/

2 http://www.broadinstitute.org/mpg/ricopili/

3 http://peana-od.inf.um.es:8080/UKBECv12/
