# Supplementary material for: A Comprehensive Analysis of the Effect of SIRT1 Variation on the Risk of Schizophrenia and Depressive Symptoms
Source: Front Genet. 2020 Jul 31;11:832. doi: 10.3389/fgene.2020.00832 (PMC7413929; doi:10.3389/fgene.2020.00832)

**Supplementary Table S1 Demographic and clinical characteristics of schizophrenia patients with or without depression for mRNA** expression analysis

| Characteristic | With depression (n=89) | Without depression (n=108) | Statistics (χ^2^) | *P* |
| --- | --- | --- | --- | --- |
| Gender M/F | 41/48 | 59/49 | 1.43 | 0.25 |
|  |  |  |  |  |
| Age (years) | 28.96±3.74 | 28.48±3.76 | 0.88 | 0.38 |
| Olanzapine duration (month) | 32.04±5.07 | 33.01±6.07 | -1.19 | 0.23 |
| Olanzapine doses (mg) | 12.28±4.84 | 11.78±4.59 | 0.73 | 0.47 |
| PANSS |  |  |  |  |
| Positive subscore | 10.02±3.10 | 10.59±3.15 | -1.28 | 0.20 |
| Negative subscore | 12.02±3.71 | 12.33±3.79 | -0.58 | 0.56 |
| General psychopathology | 22.29±4.32 | 22.87±3.92 | -0.99 | 0.33 |
| Total score | 44.34±8.16 | 45.80±8.09 | -1.25 | 0.21 |
| CDSS total scores | 8.85±1.25 | 3.46±1.56 | 26.36 | <0.01 |

Note: Data presented as*x*±s.

**Supplementary Figure S1 Association of rs3758391 with schizophrenia in PGC database**


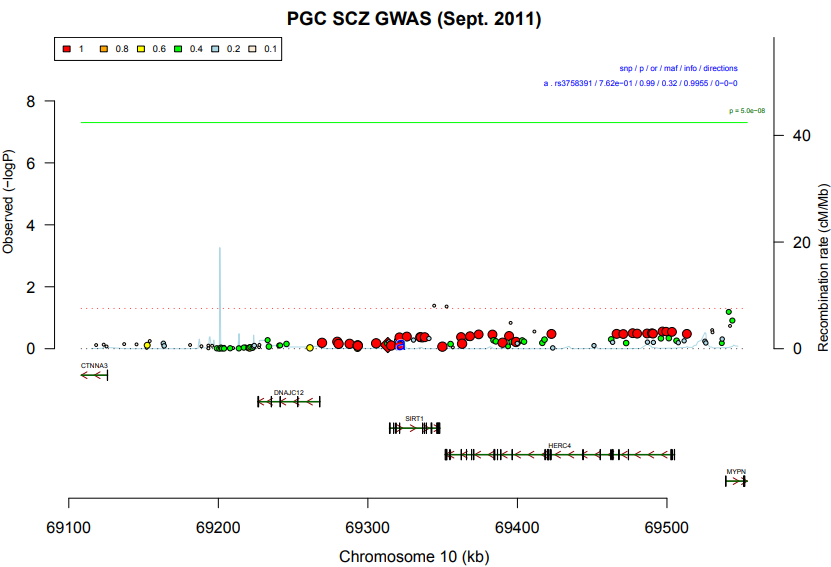

Supplement: Supplementary file 1 [file Data_Sheet_1.doc]
